# Factors Affecting the Time and Process of CMC Drying Using Refractance Window or Conductive Hydro-Drying

**DOI:** 10.3390/gels7040257

**Published:** 2021-12-11

**Authors:** Rubén D. Múnera-Tangarife, Efraín Solarte-Rodríguez, Carlos Vélez-Pasos, Claudia I. Ochoa-Martínez

**Affiliations:** 1 Grupo GIEPRONAL, School of Basic Sciences, Technology and Engineering, Universidad Nacional Abierta y a Distancia, Palmira 763531, Colombia; ruben.munera@unad.edu.co; 2 Grupo GIPAB, School of Food Engineering, Universidad del Valle, Santiago de Cali 760001, Colombia; carlos.velez@correounivalle.edu.co; 3 Quantum Optics Research Group, Department of Physics, Universidad del Valle, Santiago de Cali 760001, Colombia; efrain.solarte@correounivalle.edu.co

**Keywords:** carboxymethyl cellulose, drying, RW, CHD, radiation penetration depth

## Abstract

Intensive research on biodegradable films based on natural raw materials such as carboxymethyl cellulose (CMC) has been performed because it enables the production of transparent films with suitable barrier properties against oxygen and fats. Considering the importance of the production of this type of film at the industrial level, a scalable and continuous drying method is required. Refractance window-conductive hydro drying (RW-CHD) is a sustainable and energy-efficient method with high potential in drying this kind of compound. The objective of this study was to evaluate the factors (CMC thickness, heating water temperature, and film type) and radiation penetration depth that affect drying time and energy consumption. It was found that drying time decreased with increasing temperature and decreasing thickness. Similarly, energy consumption decreased with decreasing temperature and thickness. However, the drying time and energy consumed per unit weight of product obtained were equivalent when drying at any of the thicknesses evaluated. Film type had little effect on time and energy consumption compared to the effects of temperature and CMC thickness. The radiation penetration depth into the CMC was determined to be 1.20 ± 0.19 mm. When the thickness was close to this value, the radiation energy was better utilized, which was reflected in a higher heating rate at the beginning of drying.

## 1. Introduction

The limited access to non-renewable resources for packaging materials has turned the focus to biopolymers. In the last decade, intensive research on biodegradable films based on natural raw materials such as carboxymethyl cellulose (CMC) has been published, as it has non-toxic and non-allergic effects and produces a transparent film with suitable barrier properties against oxygen and fats [1,2]. These films are used for food packaging, adhesives, biocomposites, and hydrogel films.

Antosik et al. [1] prepared CMC-based films as carriers for a pressure-sensitive adhesive. The polysaccharide solution was poured into a polystyrene mold and dried for 24 h at 60 °C. Hasheminya et al. [2] prepared films with Kefiran, essential oil, and CMC; the final step of the process was to pour the solution onto glass plates and dry at 25 °C for 72 h. In addition, CMC was used for its binding capacity in tea waste bioplastics; the drying process took place in an oven for 3 h at 70 °C [3]. Cheng et al. [4] prepared a composite film using CMC, konjac glucomannan, and palm olein to improve the moisture barrier properties. The solutions were dried at room temperature for approximately 20 h. Li et al. [5] produced a starch-CMC-based film; samples were dried at room temperature for 12 h. Another example of based CMC films is hydrogels. They can offer new opportunities to design efficient packaging materials when prepared with CMC and polyvinylpyrrolidone [6]. These works used the casting method to dry the films at laboratory scale. Considering the importance of the production of this type of film at the industrial level, a drying method that is scalable, continuous, and energy efficient is required.

Refractance Window^®^ (RW) drying is a technique developed by MCD Technologies, Inc. (Tacoma, WA, USA) [7,8]. This technique is called conductive hydro-drying (CHD) for high thicknesses [9]. The material to be dried is placed on a film that is in contact with water (95–97 °C) at atmospheric pressure. The water is recirculated below its boiling point to provide the thermal energy necessary for drying. According to some authors, this film is transparent to infrared radiation [7,8,10]. The thermal energy of the water is transmitted to the food through the plastic film by conduction and radiation, and moisture is carried by air flowing over the food layer [9]. This technology has had substantial growth in the last five years (75% of the publications on the subject have been in this period) due to its advantages in quality and cost.

One of the advantages of RW-CHD drying is the drying time, which is relatively short when compared to other drying techniques, such as solar drying, tray drying, or freeze drying. In tray drying, the products need to be dried from 3 to 5 h at high temperature whereas the processing time in freeze drying varies from 18 to 24 h [11]. Ochoa-Martínez et al. [12] found that, for the RW technique, the moisture content decreases rapidly to a value below 5% wb in about 30 min for 1-mm samples and 60 min for 2-mm samples. In contrast, it took about 240 min to obtain similar results with the tray drying technique. Baeghbali et al. [13] obtained a drying time of 20–24 h for freeze drying whereas the observed time was 5–7 min for RW for different products. Nindo et al. [14] reported residence times (h) of drying for asparagus puree for freeze drying (18–24), tray drying (2.5–5.5), as well as spouted bed (1.2–2.3), microwave (0.5–1.6), and RW (0.074) technologies. Longer drying times adversely affect product quality. The faster drying rate in RW-CHD has been attributed to the radiative heat transfer that occurs between the heating water and the food [14,15]. In CHD, there is, additionally, a significant effect of conduction heat transfer [16].

On the other hand, compared with conventional dryers, RW dryers have high thermal efficiency (i.e., two-fold higher than freeze dryers) [8]. Baeghbali et al. [13] compared the overall energy efficiency from different drying techniques for pomegranate juice drying. The highest energy efficiency (31.56%) was shown by the RW dryer, followed by a spray dryer (12.92%) and a freeze dryer (1.12%). Additionally, Baeghbali et al. [17] reported that the overall energy used for carrot puree drying was 0.375–0.525 kW for RW dryers, which was lower than the 70–84 kW used in the case of freeze drying. Similar results were reported by Bernaert et al. [18], where the energy efficiency of RW was three-fold and 40-fold higher than spray drying and freeze drying, respectively.

In addition to the above advantages, the drying equipment, energy consumption, and operation costs are lower than those of tray, freeze, drum, and spray drying methods. Additionally, it has negligible CO_2_ emissions [19]. For example, the cost of the WR drier can be between one-third and one-half the cost of freeze drying [8,15,20]. Compared to other drying systems such as drum drying or convection drying, production costs can be up to 70% lower [21,22].

The effect of process variables (sample thickness and heating water temperature) on physicochemical properties has been studied for numerous foods, especially fruits and vegetables and, to a lesser extent, meats, dairy products, and others. Mahanti et al. [23] conducted an extensive review on this subject. Moderate food temperatures and short drying times reduce the deterioration of food characteristics. Other important process variables are the type and thickness of the film used, which affect the percentage of radiation transmission. These variables have not been studied. Most of the relevant works have used polyethylene terephthalate (PET) films. Other authors [9,16,24] performed calculations to determine the relative contribution of heat transfer mechanisms in RW-CHD drying. In no case was the penetration of infrared radiation observed, which is a critical aspect affecting heat transfer in this technology. There are no available data in the literature about this variable for infrared wavelengths, except that of Ginzburg [25] for various food materials and Almeida et al. [26] for potatoes in near-infrared drying. In addition, heat fluxes emitted by water, water–film, and water–film–CMC systems have not been measured to characterize RW-CHD drying.

In RW-CHD, comprehension of the factors affecting drying time and energy consumption, such as the thickness of the sample, the temperature of the heating water, and the type and thickness of the film, is important. Only the first two have been studied for numerous products, especially fruits and vegetables and, to a lesser extent, meats, dairy products, and others. This technology has not been applied in drying gels such as CMC. Because of its high moisture content and long processing time, it is essential to use highly efficient drying methods for the drying of CMC films that can be used as biodegradable packaging materials.

The main objective of the work was to evaluate the factors affecting the drying time and energy consumption in the RW-CHD drying of CMC. The radiation penetration depth into CMC and the effect of operating conditions (CMC thickness, heating water temperature, and film type and thickness) on drying time and energy consumption were determined.

In this work, it was found that the drying time decreased with increasing temperature and decreasing thickness. Similarly, energy consumption decreased with decreasing temperature and thickness. However, the drying time and energy input per unit weight of product obtained were equivalent when drying at 1.5 or 3.5 mm. That is important for deciding the amount of sample to be dried (product thickness) as a function of operating cost and final product quality when exposed to high temperatures for a longer time. Compared to temperature and CMC thickness, film type had a minor effect on time and energy consumption. This was due to the films’ high radiation transparency (low resistance to heat flow), regardless of type and thickness. On the other hand, the radiation penetration depth in CMC was determined to be 1.20 ± 0.19 mm. This was independent of the type and thickness of the film and the temperature of the heating water since it is a property of the material; no effect of temperature was observed because the wavelength range at the temperatures studied is very small. For thicknesses close to this value, more use is made of the radiation energy, which was reflected in a higher heating rate at the beginning of drying (3 min).

## 2. Results and Discussion

### 2.1. Drying Kinetics

**Figure 1:**
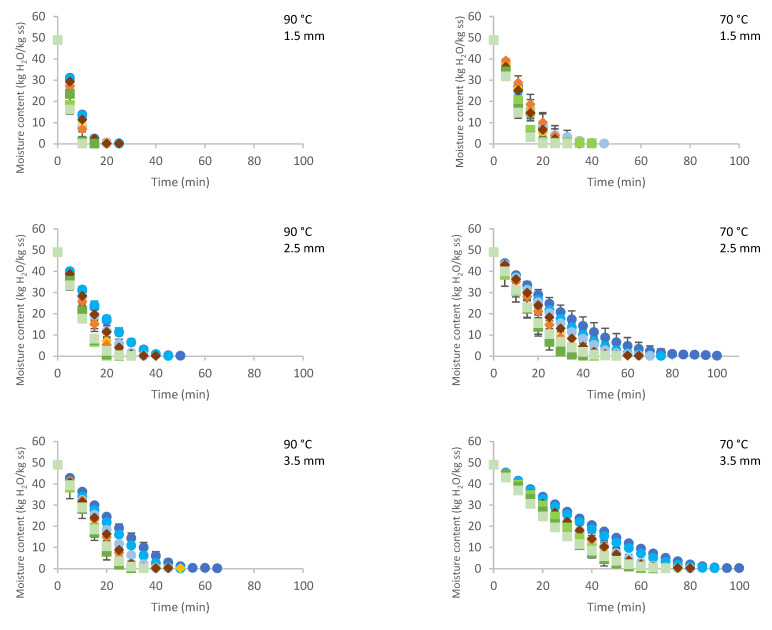
Effect of drying time, water temperature, and CMC thickness on moisture content (●PP 0.83, ●PP 0.55, ●PP 0.38, 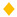 LDPE 0.15, 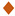 LDPE 0.10, 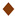 LDPE 0.03, 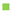 PET 0.25, 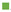 PET 0.18, 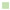 PET 0.08).

Figure 1 shows the drying kinetics of CMC with thicknesses of 1.5, 2.5, and 3.5 mm for the three films (PP, LDPE, and PET) at 90 and 70 °C. As expected, as the sample thickness decreased and the drying temperature increased, the water loss was higher for every film.

Considering that the air conditions in contact with the sample during drying were 60.4 ± 6.5% relative humidity and 26.9 ± 1.7 °C, the equilibrium moisture content for CMC is 0.17 kg water/kg dry solid (15% wb) according to the sorption isotherm reported by Torres et al. [27]. In the kinetics shown, values close to this equilibrium value can be observed.

**Figure 2:**
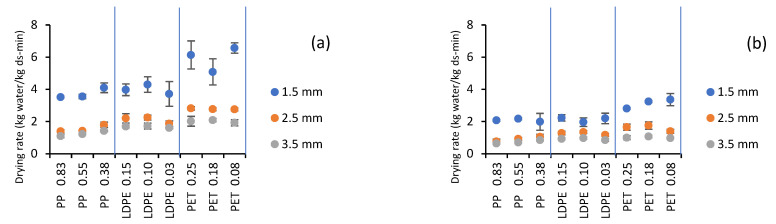
Effect of type and thickness of plastic films and CMC thickness on the drying rate: (**a**) 90 °C and (**b**) 70 °C.

An initial part with a constant slope was observed in these curves, corresponding to the constant drying rate period, up to an approximate moisture value of 5 kg water/kg ss (83% wb). The high drying rate in this period was notorious, unlike tray drying, which is very short with a low drying rate [12]. During this period, there was water saturation on the surface and easy replacement of evaporated water due to the low resistance to mass transfer in the interior. In RW-CHD, the sample exhibited rapid heating (in the first seconds of drying), maintaining the continuous transport of water inside the sample to the surface, in contrast to the tray drying, where the sample heating was slow, and heat transfer was the limiting mechanism. Moisture removal from the surface to the air occurred by convective diffusion, the driving force of which is the water concentration difference. Parrouffe et al. [28] found that infrared radiation had no significant influence on reducing drying time beyond the constant rate period due to the low moisture content at the surface, which generally has a low penetration depth or a low absorption. The drying rate in the constant rate period (kg water/kg ss-min) corresponding to the slope of the curves in the linear region is presented in Figure 2.

In the analysis of variance, it was observed that the main factors (type-film thickness, CMC thickness, and heating water temperature) and their interactions had a significant effect on the drying rate (*p* < 0.05). According to the F-Ratio value, the relative importance of CMC thickness (1548.63) related to temperature (429.83) and film type-thickness (73.59) was highlighted. Tukey tests for temperature, CMC thickness, and film thickness type are presented in Table 1, Table 2 and Table 3.

According to the Tukey analysis presented in Table 1, the mean value of the drying rate was significantly different for the three temperatures and the three CMC thicknesses studied. The rate increased with increasing heating water temperature and decreasing CMC thickness (higher values were obtained for a CMC thickness of 1.5 mm) and higher differences for PET films. The mean value of the drying rate increased by 81.7% when increasing the temperature from 70 to 90 °C and was 177.0% when decreasing the CMC thickness from 3.5 to 1.5 mm. Similar behavior was observed by Zotarelli et al. [24] in mango drying, with an increase of 40% when decreasing the thickness from 2 to 3 mm.

Table 2 shows that the three thicknesses of each film evaluated had a similar mean drying rate value. The highest rate was obtained for the PET films, while the other two materials presented lower values that were statistically equal.

Although the tests with PET for a sample thickness of 1.5 mm showed a higher drying rate in the constant period (moisture content dropped from 98% to 80% wb), statistical analysis of the results showed that the film type had little effect on time compared to the temperature effect and CMC thickness. According to Tsilingiris [29], a slight change in chemical composition, thickness, and measurement processes should produce substantial changes in the optical properties of the film.

**Figure 3:**
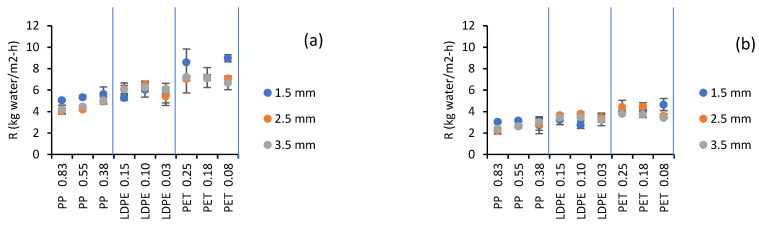
Effect of type and thickness of plastic films and CMC thickness on the drying flux (R): (**a**) 90 °C and (**b**) 70 °C.

Figure 3 shows the drying flux, which corresponds to the water evaporation capacity of the equipment per unit of drying area (kg water/h-m^2^). These values were between 2 and 9 kg/h-m^2^, similar to those obtained by Zotarelli et al. [24] (between 2.67 and 10.75 kg/h-m^2^) when drying mango pulp (thickness of 2, 3, and 5 mm) and heating water of 75, 85 and 95 °C. In addition, Nindo et al. [14] obtained a value of 10 kg/h-m^2^ when drying pumpkin puree, with thicknesses between 0.4 and 0.6 mm in the pilot plant and of 3.1, 3.9, and 4.6 kg/h-m^2^ at the industrial level.

All factors and their interactions affected the drying flux (*p* < 0.5). According to the F-ratio, the water temperature (700.62) had the most significant effect. Film thickness (92.80) and CMC thickness (17.84) had far less significant effects. According to Tukey’s test (Table 1), the three temperatures had effects that differed in terms of significance. The mean value of the drying flux increased with increasing temperature. A similar effect was observed in the drying of mango pulp [24]. By increasing the temperature from 70 to 90 °C, the drying flux increased by 79%.

On the other hand, Tukey’s test for CMC thickness (Table 2) showed that the mean value of the drying flux was higher and statistically different for CMC thickness of 1.5 mm. The mean drying flux obtained with 2.5 and 3.5 mm CMC thicknesses was not significantly different. The mean drying flux value increased by 9.0% when reducing the thickness from 3.5 to 1.5 mm. Zotarelli et al. [24] observed similar behavior when decreasing the thickness of mango pulp.

The Tukey test (Table 3) for film type and thickness showed that the mean value of the drying flux was statistically similar for the three thicknesses of each of the films and different for each type of film. The highest drying flux was obtained with PET, followed by LDPE and PP.

**Figure 4:**
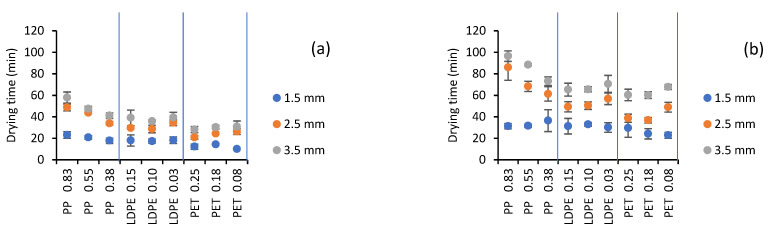
Effect of type and thickness of plastic films and CMC thickness on the drying time: (**a**) 90 °C and (**b**) 70 °C.

Figure 4 shows the drying time required to reach a final moisture content of 0.312 kg water/kg ss (this corresponds to the highest final moisture content of all treatments). Statistically, it was observed that all factors had a significant effect (*p* < 0.05) on drying time (min). According to the F-ratio, the most significant effect was CMC thickness (993.90), followed by temperature (540.26). Shende and Datta [30] showed the same behavior for mango drying. Film type-thickness (93.34) showed a minimal effect. Azizi et al. [31] also found that increasing the thickness of PET film from 0.1 to 0.3 mm does not have any significant effect on the drying time of kiwifruit irrespective of water temperature and sample thickness.

In studies of fruit drying using PET, drying times that were comparable to those presented in Figure 4 were obtained. For cornelian cherry pulp with a thickness of 1 mm at 90, 95, and 98 °C, the drying time was between 15 and 20 min [32]; a time of 20 min was obtained for mango pulp with a thickness of 2 mm at 95 °C [30]; and a time of 40 min was recorded for tomato pulp with a thickness between 1 and 1.5 mm at 90 °C and 60 min at 75 °C [22]. The increase in drying time with increasing food thickness was also observed in the drying of papaya puree [33] and mango slices [12].

According to Tukey’s test, significantly different means were obtained for the temperatures and CMC thicknesses evaluated (Table 1 and Table 2). As expected, the mean value of drying time decreased with increasing temperature and decreasing thickness. A decrease from 90 to 70 °C increased the drying time by 78.6%; an increase in thickness from 1.5 to 3.5 mm increased the drying time by 138.1%. However, there was a direct relationship between time and the amount of final product obtained, which in turn was related to CMC thickness (R^2^ = 0.9992) (Table 4), meaning that it was equivalent to drying with 1.5 or 3.5 mm in terms of the final amount obtained. This is important for deciding on the amount of sample to dry in terms of final product quality and operating costs. According to Table 3, for PP, the mean value of drying time was significantly different for each of its thicknesses and higher than for the other films evaluated (the evaluated thicknesses of PP were very high compared to those of LDPE and PET). The lowest average value of the drying time was obtained with the PET films (regardless of their thickness), which agrees with the higher drying rate obtained.

When the yield analysis (g/h-m^2^) was conducted for the conditions studied (Table 5), it was observed that the highest values were obtained for 3.5 mm food thickness and 90 °C. The highest yield was obtained for PET 0.25. The best performances for LDPE and PP were observed with LDPE 0.1 and PP 0.38, respectively. In addition to the yield, it was necessary to consider the temperatures to which the product was subjected during drying, which affected the quality of the final product.

### 2.2. Temperature Profiles

**Figure 5:**
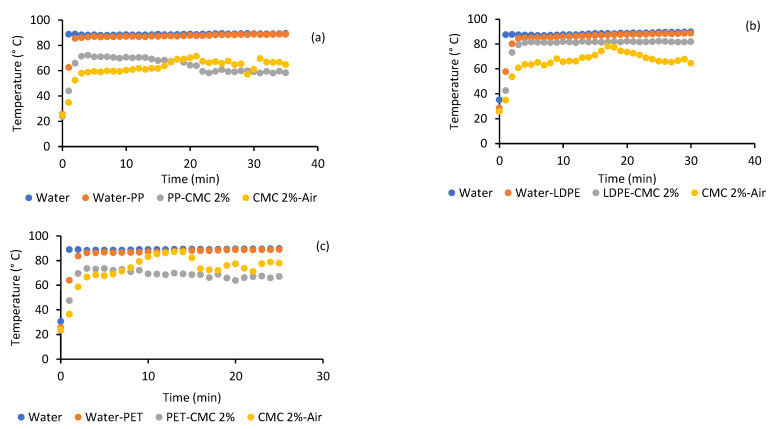
Temperature profiles of CMC-2.5 mm drying at water temperature of 90 °C: (**a**) PP 0.38 mm, (**b**) LDPE 0.15 mm, and (**c**) PET 0.18 mm.

Figure 5 shows the temperature profiles of the main components of the system (water, water–film interface, film–CMC interface, and CMC–air interface) for some of the conditions studied.

The interfaces of PP-CMC and PET-CMC films were rapidly heated by radiation and conduction (within the first three minutes of drying) to approximately 10–20 °C below the source temperature. During drying, their temperature decreased by 5–10 °C as the CMC absorbed energy from the film to equilibrate. In general, they may not heat up to the source temperature due to their high transmissivity (70–90 %) and low conductivity (PP: 0.23–0.26 W/mK; PET: 0.16–0.20 W/mK) [34]. On the other hand, when using LDPE, the interface temperature with CMC increased rapidly. Due to its high conductivity (0.25–0.33 W/mK) [34], it reached a value close to the heating water (about 5 °C below the water temperature) and remained constant during drying. The film interface temperature that did not depend on the food was always constant, while that which was in contact with the food was a function of the food temperature.

The energy absorbed by the sample was used to heat (sensible heat) and to evaporate the water (latent heat). In general, the CMC temperature remained below the water temperature due to evaporative cooling and the effect exerted by the air temperature (26.9 ± 1.7 °C). On the other hand, the CMC initially absorbed energy from the film and warmed up to equilibrium with the temperature of the film–CMC interface (the film lost energy and the food gained energy); subsequently, heating of the sample was observed, and finally, they equilibrated at the same temperature. An increase in the sample temperature up to water temperature was only observed in the drying with LDPE-0.03 due to the low film thickness and high conductivity. The sample temperature rose to that of the water in tests carried out at 70 °C with 1.5 mm thickness, regardless of the film used.

### 2.3. Heat Flux Emitted by Water, Water–Film, and Water–Film–CMC Systems

**Figure 6:**
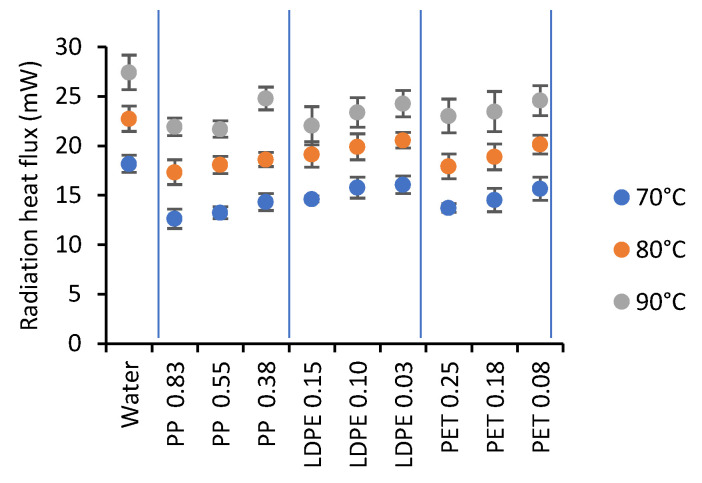
Radiation heat transfer for water and water-drying film systems.

The radiative heat flux emitted by the water at 70, 80, and 90 °C and the radiative heat flux transmitted through the film (water–film system) determined during the first 50 s were measured (Figure 6). It was observed that the heat flux was significantly higher for times longer than 50 s (these data are not presented). The heating of the film after this time (Figure 5) increased its emissivity, causing an increase in heat flux. Statistically, a high-temperature effect was observed.

According to Figure 6, all films showed high transmittance to radiation, with the heat flux being similar to that of the water. These results agree with those presented by Tsilingiris [29] for PP and LDPE films. That confirms that the films were transparent to radiation to a high degree, which allowed the heating of the sample, as discussed in the previous section.

**Figure 7:**
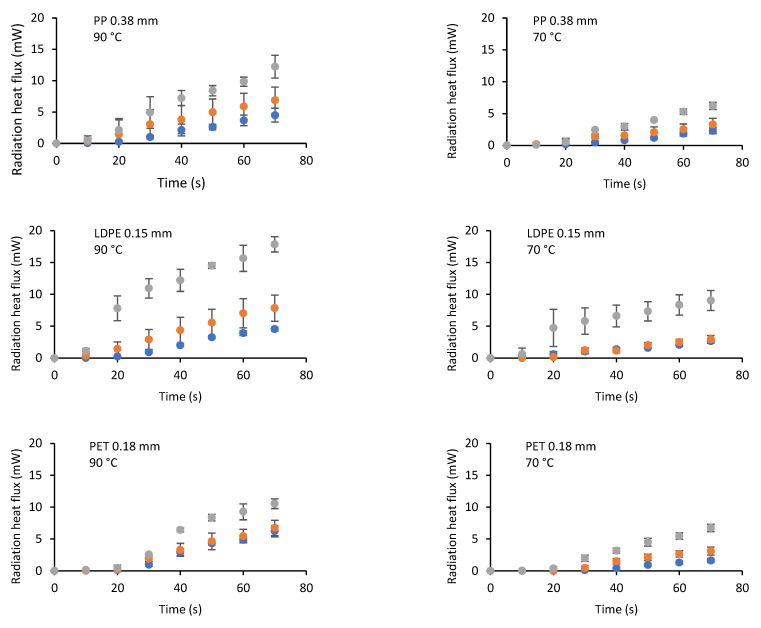
Effect of the type of plastic film, water temperature, and CMC thickness (●1.5 mm, ●2.5 mm y ●3.5 mm) on the radiation heat flux leaving the CMC.

On the other hand, the radiation heat flux leaving the CMC during the first 70 s of drying is presented in Figure 7 for some of the conditions studied. It was observed that the radiative heat flux was higher for heating water at 90 °C when compared to 80 or 70 °C. It was also observed that it increased with time; initially, this heat flux was low due to the high radiation absorption of the food; subsequently, the flux increased due to the combined effect of the radiation emission of the food upon heating and the lower absorption.

### 2.4. Penetration Depth of the Infrared Radiation in CMC

The penetration depth is defined as the depth at which the radiation intensity decays by 37% (1/e) of its initial value [35,36]. In other words, 63% of the incident radiation is used [11,30]. Depending on the material, electromagnetic waves can travel inside the food or be absorbed on its surface [37]. Using (Equation (1)), the parameters $I_{0}$ and $\mu^{-1}\;$ were obtained (Table 6).

(1) $$I=I_{0}e^{-\mu x}$$

where *I* is the radiation passing through the solid (water–film–food system) of thickness *x*, $I_{0}$ is the initial radiation reaching the solid, and *µ* is the attenuation coefficient.

The ANOVA and Tukey’s test showed no significant effect of temperature or film type-thickness, so the values obtained for all penetration depths were averaged (Table 6). The average value of the radiation penetration depth in 2% CMC was 1.20 ± 0.19 mm. Although the penetration depth is known to depend on the nature of the material and temperature of the emitting source (wavelength) [37], the value obtained depended only on the material (CMC 2%) in the range of temperatures evaluated (70, 80, and 90 °C). It was, as expected, independent of the type and thickness of the films evaluated.

The total emissive power includes the energy of all wavelengths in the radiation spectrum [37]. The wavelength at which the maximum emissive power of radiation occurs, $\lambda_{max}$, depends on the emitter temperature (*T*, K) and is given by Wien’s displacement law, presented in Equation (2) [38].

(2) $$\lambda_{max}=\frac{2898}{T}$$

According to Equation (2), for heating water temperatures between 70 and 90 °C, the wavelengths at which the maximum emissive power is obtained are 8.4 µm and 8.0 µm, respectively. This slight difference in wavelengths for the studied temperature range explains the lack of an effect of this factor on the penetration depth.

There is little information available in the literature on the penetration depth of infrared radiation [26,37]. For foods with high humidity (>80), penetration depth values between 1 and 7 mm were found, depending on the spectral peaks of the radiation source [25]. For potatoes with humidity between 67 and 82%, radiation penetration depth values between 0.45 and 2.85 mm were obtained [26], and for carrots, 1.5 mm was reported [25].

The amount of infrared radiation that a vegetable or animal sample can absorb depends on the wavelength of the emitter and the sample composition [37]. Foods are complex mixtures of biochemical molecules, biological polymers, inorganic salts, and water. Water shows high absorption at wavelengths of 3.0, 4.7, 6.0, and between 12 and 15.3 µm [39]. These values do not coincide in the wavelength range where the highest emissive power of water is obtained (8.4 µm and 8.0 µm at the temperatures used). On the other hand, carbohydrates (i.e., CMC) show two strong absorption bands at 3 µm and between 7 and 10 µm [39], coinciding with the emission bands of water. Accordingly, resonant absorption occurs in carbohydrates, which heat the water in the CMC. Subsequently, a thermal heating effect of the water occurs due to the dipole orientation in the electromagnetic field of radiation.

### 2.5. Energy Consumption

**Figure 8:**
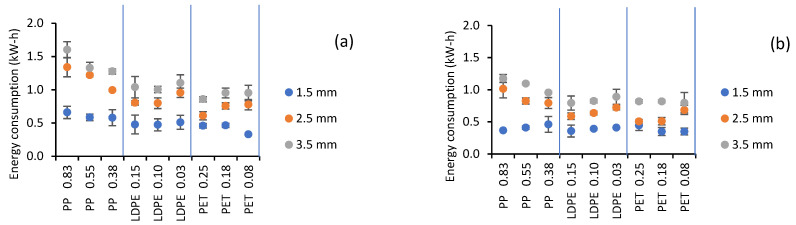
Effects of type and thickness of plastic films, CMC thickness, and water temperature ((**a**) 90 °C and (**b**) 70 °C) on energy consumption.

Figure 8 shows the results of energy consumption. According to the analysis of variance, film thickness, CMC thickness, and the heating water temperature had a significant effect on energy consumption (*p* < 0.05). According to the F-ratio, the most significant factor was CMC thickness (907.56), while the heating water temperature (101.8) and film thickness (72.56) had a much smaller effect.

Tukey’s test showed significant differences for both thickness and temperature. Energy consumption increased by 125.0%, thereby increasing the CMC thickness (*p* < 0.05) from 1.5 to 3.5 and 26.9% with the increase in temperature from 70 to 90 °C. This behavior was expected considering the greater amount of water to evaporate at the higher thickness and the more significant amount of energy required with increasing temperature.

The Tukey test for film thickness and type showed that the mean energy consumption value was higher for PP 0.83 mm, followed by PP 0.55 and 0.38 mm. There was no significant difference in the mean value of energy consumption (*p* < 0.05) among the other films, probably due to a combined effect of transmissivity and conductivity.

In the present work, the energy consumption varied between 0.44 and 0.99 kWh depending on the thickness of the sample. On the other hand, Baeghbali et al. [13] reported a consumption of 4.31 ± 0.82 kWh (using a pilot-scale continuous dryer in the drying of pomegranate juice). When analyzing the specific energy consumption ratio (SER) [19], which in turn was related to the CMC thickness (Table 7), a direct relationship was found (R^2^ = 0.9989). This means that the energy consumption, in terms of the final amount of product obtained for the three thicknesses studied, was similar. As mentioned in the time calculation, this information is essential for deciding on the amount of sample to dry depending on the final product quality and operating costs. Menon et al. [19], in their review of energy efficiency in drying technologies, reported SER values between 0.13 and 1.9 kWh/kg for super-heated steam drying, between 0.7 and 37.1 kWh/kg for microwave drying, and between 1.41 and 3.11 kWh/kg for impinging heat drying.

## 3. Materials and Methods

### 3.1. Carboxymethylcellulose (CMC)

CMC powder (Gelycel F1 3500, specification 10031) with 4.14% humidity (Agenquímicos, Santiago de Cali, Colombia) was used. It was diluted in distilled water to obtain a 2% solution (98% moisture) using a blender (Samurai, Innova, Colombia) until a uniform mixture was obtained. The solution was left at rest for 24 h to release all air bubbles. Metal frames of 9.5 cm × 29.5 cm were used to obtain CMC sheets of the desired thickness (1.5, 2.5, and 3.5 mm).

### 3.2. Equipment

**Figure 9:**
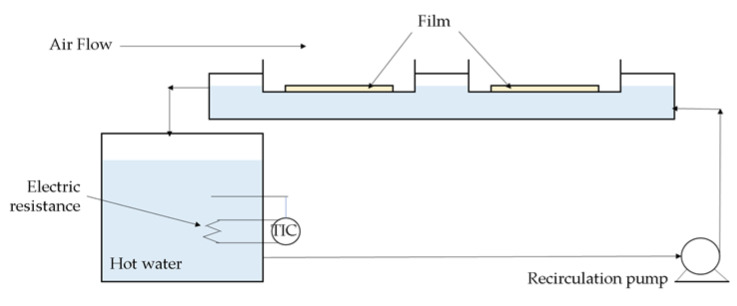
Schematic diagram of an RW-CHD dryer.

A stationary RW-CHD dryer (HS-50, Ceirobots, Santiago de Cali, Colombia) (Figure 9) was used. Two drying trays (10.3 cm × 30.3 cm), the bottoms of which were composed of plastic film, were in contact with the hot water. CMC was placed on the plastic films. The water was heated by two resistors (14.7 A each) in a 20 L tank, and the temperature was maintained through a recirculation system. A fan was used to remove the water vapor produced during drying. Three films of different thicknesses were evaluated, each according to commercial availability: polypropylene (PP) of 0.83, 0.55, and 0.38 mm; low density polyethylene (LDPE) of 0.15, 0.10, and 0.03 mm; and polyethylene terephthalate (PET) of 0.25, 0.18, and 0.08 mm.

### 3.3. Experimental Design

The factors evaluated were the type of film with different thicknesses (PP-0.83, PP-0.55, PP-0.38, LDPE-0.15, LDPE-0.10, LDPE-0.03, PET-0.25, PET-0.18, PET-0.08), the thickness of the CMC (1.5, 2.5, and 3.5 mm), and the temperature of the heating water (70, 80, and 90 °C). A 9 × 3 × 3 complete randomized factorial design was used. All tests were performed in triplicate for a total of 243 treatments. The response variables evaluated were moisture content and drying time, temperature profiles, radiation heat flux, radiation penetration depth, and energy consumption. In addition, the radiant heat flux for the water and water–film system was determined at 70, 80, and 90 °C.

### 3.4. Response Variables

#### 3.4.1. Moisture Content and Drying Time

Moisture content (db) was determined every 5 min. The drying tray was re-removed from the dryer, and the bottom dried and weighed. A balance (Ohaus^®^, Ad-venturerTM, Shanghai, China, accurate to 0.01 g) was used. The initial and final moisture content of the sample was determined by oven drying (Thermo Scientific, Heratherm OGS60, Langenselbold Germany) at 70 °C for 24 h [32]. The drying time required to reach a moisture content of 0.312 kg water/kg ss of CMC was determined to compare treatments (this value was selected considering that it was the minimum value obtained in some treatments).

#### 3.4.2. Temperature Profiles

The temperatures of the hot water, the water–film interface, and the film-water-food interface were measured every 5 s using J-type thermocouples (Omega, 5TC-TT-J-30-72, diameter 0.25 mm, Norwalk, CT, USA) and a data acquisition system (Comark Instruments, model Diligence Evg N3014, Norwich, UK), with an accuracy of 0.2 °C. A thermographic camera (Flir, Model E4, Wilsonville, OR, USA) was used to measure the temperature of the food–air interface.

#### 3.4.3. Radiative Heat Flow

The radiative heat flux emitted by the water, the water–film system, and the water–film–food system was measured for all experimental design conditions. A potentiometer (Molectron Detector Inc., PowerMax 5200, Portland, OR, USA) with a radiation measurement scale between 0 and 10 W (12 scale ranges; the scale from 0 to 100 mW was used) and an accuracy of 0.1 mW was used. This equipment used a pyroelectric sensor to measure the radiation heat flux in a spectral range of 0.25 to 11 µm (from ultraviolet to infrared). The diameter of the measuring sensor was 19 mm. The sensor was placed 4 cm from the radiant-heat-flux-emitting surface. The measurements were performed before the heating of the film or CMC, and the radiative heat flux was calculated by dividing the heat flux by the sensor area (0.000284 m^2^). Measurements were taken between 30 and 70 s. Before 30 s, the system had not stabilized, and after 80 s, conduction heat transfer was likely to occur due to heating of the film. All these measurements were taken on the drying film without food.

#### 3.4.4. Radiation Penetration Depth

The radiation penetration depth ($\mu^{-1}\;$or *x*_0.37_) is defined as the depth at which the radiation intensity decays by 37% (1/e) of its initial value [35,36]. Using the radiation attenuation law, also known as the Beer–Lambert Law [29,40] (Equation (1)), the values of the intercept ($ln\left(I_{0}\right)$) and the slope (−*μ*) were determined. For this, the experimental values of *I* vs. *x* (1.5-, 2.5-, and 3.5-mm thickness CMC, for heating water at 70, 80, and 90 °C) were used.

The radiation penetration depth was calculated for a radiation heat flux at 30 s, thus considering only the radiation emitted by the hot water passing through the film and the food. Data corresponding to 10 and 20 s were not considered due to the system’s instability at the beginning of the measurement.

#### 3.4.5. Energy Consumption

Energy consumption was determined using a Peacefair meter, model PZEM-022 (China), for alternating current in the voltage range of 80 to 260 V, 50/60 Hz, up to 100 A. The sensing element of this equipment was placed at the power input of the drying equipment.

### 3.5. Statistical Analysis

Statgraphics Centurion 19 software (Statgraphics Technologies, Inc., version 19.2.01, 2021, The Plains, VA, USA) was used to perform analysis of variance and determine the dependence between process variables and response variables, with a 95% confidence level (*p* < 0.05). It was also used to determine the independence between means using Tukey’s test.

## Figures and Tables

**Table 1:** Effect of water temperature on CMC drying kinetics.

Temperature (°C)	Drying Rate (kg Water/kg ds-min)	Drying Flux (kg Water/h-m^2^)	Time (min)
70	1.53 ^a^	3.38 ^a^	52.37 ^a^
80	2.14 ^b^	4.73 ^b^	37.52 ^b^
90	2.78 ^c^	6.05 ^c^	29.33 ^c^

Values followed by the same letter are not significantly different (*p* < 0.05, Tukey’s test).

**Table 2:** Effect of CMC thickness on drying kinetics.

CMC Thickness (mm)	Drying Rate (kg Water/kg ds-min)	Drying Flux (kg Water/h-m^2^)	Time (min)
1.5	3.49 ^a^	4.96 ^a^	22.67 ^a^
2.5	1.70 ^b^	4.66 ^b^	42.58 ^b^
3.5	1.26 ^c^	4.55 ^b^	53.97 ^c^

Values followed by the same letter are not significantly different (*p* < 0.05, Tukey’s test).

**Table 3:** Effect of type and thickness of plastic film on CMC drying kinetics.

Film Type-Thickness (mm)	Drying Rate (kg Water/kg ds-min)	Drying Flux (kg Water/h-m^2^)	Time (min)
PP 0.83	1.55 ^d^	3.46 ^e^	55.86 ^a^
PP 0.55	1.70 ^cd^	3.75 ^de^	48.85 ^b^
PP 0.38	1.90 ^bcd^	4.13 ^cd^	42.80 ^c^
LDPE 0.15	2.06 ^bc^	4.68 ^b^	38.31 ^cd^
LDPE 0.10	2.10 ^b^	4.85 ^b^	37.66 ^cd^
LDPE 0.03	1.93 ^bcd^	4.59 ^bc^	39.42 ^cd^
PET 0.25	2.68 ^a^	5.77 ^a^	30.36 ^e^
PET 0.18	2.66 ^a^	5.62 ^a^	30.56 ^e^
PET 0.08	2.76 ^a^	5.64 ^a^	33.86 ^de^

Values followed by the same letter are not significantly different (*p* < 0.05, Tukey’s test).

**Table 4:** Relation between the dry product and the sample thickness.

Thickness (mm)	Drying Time (min)	Wet Sample Weight (g)	Dry Sample Weight (g)	Drying Index (g Dry Sample/h)
1.5	22.7	33.3 ± 1.2	0.9 ± 0.0	2.6 ± 0.9
2.5	42.6	64.8 ± 3.8	1.7 ± 0.1	2.6 ± 0.8
3.5	54.0	84.9 ± 3.0	2.2 ± 0.1	2.7 ± 0.8

**Table 5:** Effects of process factors on the drying yields (g/h-m^2^).

Temperature (°C)	70			80			90		
Thickness (mm)	1.5	2.5	3.5	1.5	2.5	3.5	1.5	2.5	3.5
PP 0.83	61.5	45.2	51.0	62.2	66.3	70.4	81.8	78.3	86.5
PP 0.55	60.4	54.7	55.9	89.3	72.4	73.1	95.0	89.1	101.5
PP 0.38	49.1	58.0	64.1	83.3	80.2	88.1	101.2	107.0	113.1
LDPE 0.15	60.7	76.0	74.1	72.1	96.4	99.8	97.8	123.0	120.8
LDPE 0.10	56.5	73.6	71.5	80.1	97.2	106.0	106.4	132.9	132.6
LDPE 0.03	64.8	68.5	70.3	94.8	105.4	106.2	108.8	110.8	126.6
PET 0.25	62.1	90.8	83.7	122.0	120.6	120.2	150.7	157.2	165.7
PET 0.18	73.9	91.0	75.8	118.2	123.8	105.2	129.5	139.8	147.2
PET 0.08	79.7	69.3	67.9	125.3	93.1	95.3	180.5	128.6	148.6

**Table 6:** Beer–Lambert Law parameters for radiant heat flow.

Water Temperature (°C)	70			80			90		
Film Type-Thickness (mm)	$\mu^{-1}\;$	*Ln (Io)*	*R* ^2^	$\mu^{-1}\;$	*Ln (Io)*	*R* ^2^	$\mu^{-1}\;$	*Ln (Io)*	*R* ^2^
PP 0.83	1.33	2.35	0.94	1.35	2.69	0.96	0.73	1.37	1.00
PP 0.55	1.10	2.54	1.00	1.34	2.78	0.96	0.83	1.20	0.99
PP 0.38	1.05	2.57	0.98	0.73	3.04	0.99	0.89	1.13	0.98
LDPE 0.15	1.21	2.71	0.93	1.31	2.91	1.00	0.93	1.08	0.95
LDPE 0.10	1.22	2.79	0.99	1.23	2.97	1.00	0.74	1.36	0.98
LDPE 0.03	1.26	2.69	0.96	1.27	2.96	0.99	0.66	1.52	0.94
PET 0.25	1.33	2.33	0.91	1.41	2.73	0.96	0.88	1.13	0.97
PET 0.18	0.70	2.73	1.00	0.95	2.71	0.97	0.84	1.19	0.86
PET 0.08	1.16	2.67	0.99	1.33	2.94	0.99	0.80	1.25	0.98

**Table 7:** Specific energy consumption (SER).

CMC Thickness (mm)	Energy Consumption (kWh)	SER (kWh/kg Water)
1.5	0.44 ± 0.09	13.6 ± 2.8
2.5	0.79 ± 0.21	12.5 ± 2.8
3.5	0.99 ± 0.20	12.0 ± 2.3

## Data Availability

The data supporting reported results can be found in Múnera-Tangarife, R.D. (2021) “Evaluación de los factores que afectan la transferencia de calor en el secado por ventana de refractancia-hidrosecado” (Ph.D. thesis, Universidad del Valle, Colombia), or they are available upon request from the corresponding author.
